# Improved analysis method of neuromuscular junction in *Drosophila* larvae by transmission electron microscopy

**DOI:** 10.1007/s12565-021-00635-6

**Published:** 2021-10-18

**Authors:** Gan Guangming, Chen Mei, Zhang Chenchen, Xie Wei, Geng Junhua

**Affiliations:** 1 School of Medicine, Southeast University, Dingjiaqiao, Gu Lou District, Nanjing, 210009 Jiangsu China; 2 The Key Laboratory of Developmental Genes and Human Disease, Southeast University, Nanjing, 210009 Jiangsu China; 3 School of Life Science and Technology, Southeast University, Nanjing, 210009 Jiangsu China

**Keywords:** *Drosophila*, Neuromuscular junction, Sheet embedding, Transmission electron microscopy

## Abstract

The *Drosophila* neuromuscular junction is an excellent model for neuroscience research. However, the distribution of neuromuscular junctions is very diffuse, and it is not easy to accurately locate during ultrathin sectioning, which seriously interferes with the ultrastructural analysis under electron microscopy that only has a small field of view. Here, we reported an efficient method for acquiring the ultrastructural picture of neuromuscular junctions in *Drosophila* larva under electron microscopy. The procedure was as follows: first, the larval sample of body wall muscle was placed between the metal mesh and was dehydrated with alcohol and infiltrated with epoxy resin to prevent the sample from curling or bending, after it was dissected and fixed into thin slices. Second, the sample was embedded in resin into a flat sheet to facilitate the positioning of the muscles. Third, carefully and gradually remove the excess resin and the cuticle of the larvae, cut off both ends of the special body segment, and trim the excess specific muscles according to the recommended ratio of trimming muscles, which would reduce the workload exponentially. At last, the trimmed sample were prepared into serial about 1000 ultrathin sections that was about total 80 microns thickness, and 30–40 sections were gathered into a grid to stain with lead citrate and uranyl acetate. This method could also be applied to the other small and thin samples such as the *Drosophila* embryo, ventral nerve cord and brain.

## Introduction

*Drosophila* is a classic model animal, its neuromuscular junction (NMJ) is also a well-known neuroscience research model, which is widely used in many fields such as neurological diseases (Ashley et al. 2018; Banerjee et al. 2017), neurodevelopment (Belalcazar et al. 2021; Ramesh et al. 2021; Titus et al. 2021; Wang et al. 2021), neurodegeneration (Johnson et al. 2021), and neurosignal transmission (Krick et al. 2021; Metwally et al. 2021; Sidisky et al. 2021). *Drosophila* larval NMJ boutons are classified by three types synaptic boutons (types I, II, and III) (Guangming et al. 2020a; Jia et al. 1993), and they scattered in the shallow layers of muscles. The type I boutons (including type Ib and type Is) in the 6th/7th and 4th muscles are very suitable for the analysis of quantity and morphology by laser scanning confocal microscopy (Ashley et al. 2018; Featherstone et al. 2002). However, the diffuse NMJ boutons are not easy to observe via transmission electron microscopy (TEM), in which the visual range is extremely narrow. Therefore, due to boutons quantity, most studies consider the analysis of the ultrastructure of type I boutons in the 6th/7th muscles at segments A_2_ or A_3_ as an efficient strategy (Atwood et al. 1993; Jia et al. 1993).

The classic protocols for TEM of *Drosophila* recommend positioning type I boutons with half-thin slices and ultrathin sections (McDonald et al. 2012). Because type Ib boutons are 3–5 μm in diameter (Atwood et al. 1993), it is easy to lose quite a few part of the type Ib boutons after half-thin slice positioning, and it is not easy to observe the central section of type Ib boutons in TEM. Therefore, data from more than 8 central type Ib boutons in 3 animals should be considered valuable; notably, the total number of type Ib boutons in the 6th/7th muscles at segment A_2_ or A_3_ was greater than 40. Type I boutons are distributed along the length of the muscle, and longitudinal sections have been widely used for reducing the workload, except for the few early studies that used crosscutting (Budnik et al. 1990).

However, the fixed muscular sample of *Drosophila* larvae is very thin and easily curled in the process of alcohol/acetone dehydration, which is more unfavorable for positioning muscle and NMJ boutons. Some papers used the shortened samples to reduce curl (Banerjee et al. 2017), but it still did not prevent the sample from bending. Here, we reported a processing procedure to obtain a completely flat muscular sample, and easy to position NMJ boutons.

## Materials and methods of TEM

The *Drosophila* melanogaster white mutant *W*^*1118*^ strain was used as the wild-type control in this study, and it was cultured in standard medium at 25 °C. The materials for sample preparation via dehydration and embedding included the following: flat-bottomed glass (or plastic) test tubes of 2.5 cm in diameter, 18 steel mesh (copper or stainless steel net), bottle stopper or sponge plug with a flat test tube, and polyvinyl film (0.2 mm thick).

### Dissection, fixation and pretreatment of the samples

**Fig. 1:**
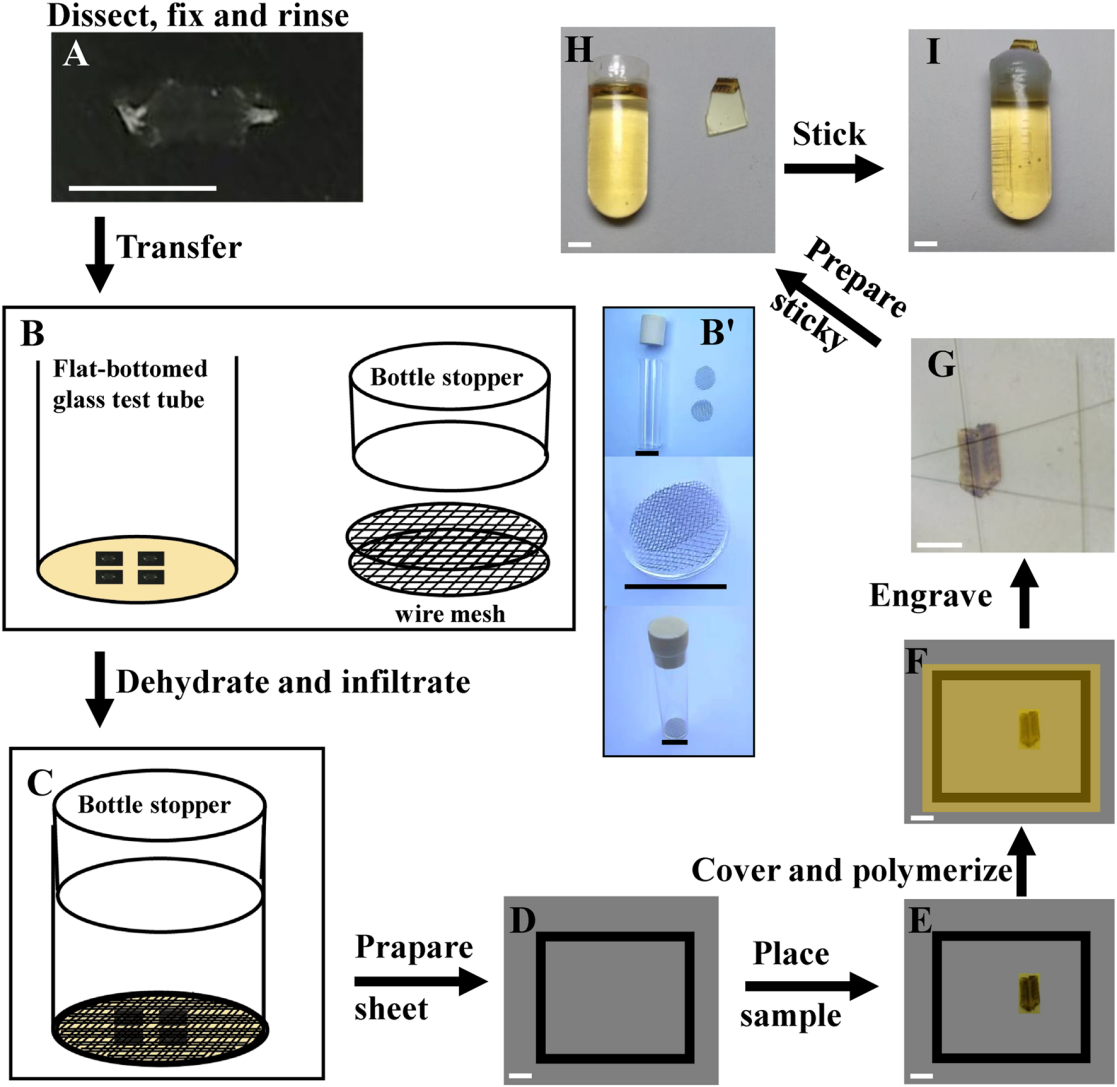
Schematic view of the preparation of the body wall muscle from *Drosophila* larvae. Traditional methods for dissection, fixation and rinsing (**A**). The sample is transferred to a homemade container (**B**, and **B’** that is an actual pictures of **B**), and is held down between the metal meshes, and then dehydrated and infiltrated (**C**). The sample is transferred to a homemade embedding tray (**D**, **E**), and be embedded and polymerized (**F**). The sample is then engraved (**G**), and placed and stick in a capsule with A/B glue (**H**, **I**). Scale bar, **A**: 4 mm; **B’**: 2.5 cm; **D**–**G**: 2 mm; **H**, **I**: 3 mm

Dissection and fixation were based on standard procedures. In brief, the wandering late-3rd-instar larvae were dissected with standard techniques in Jan solution (128 mM NaCl, 2 mM KCl, 4 mM MgCl_2_, 35 mM sucrose, 5 mM HEPES, pH 7.4) and fixed at 4 °C overnight in a mixture of 2% glutaraldehyde and 2% formaldehyde in 0.1 M sodium cacodylate buffer (pH 7.4), followed by several rinses with cacodylate buffer (Fig. 1A). The samples were then postfixed for 2 h with 1% OsO_4_ in cacodylate buffer and rinsed twice with distilled water. The preparations were stained for 2 h with 2% saturated uranyl acetate in distilled water and rinsed twice with distilled water.

### Transfer, dehydration and infiltration

The following procedure was used for epoxy resin embedding. A wire net was placed in the bottom of a flat-bottomed glass test tube, samples for TEM were transferred onto the wire net with the muscle side up, and then the wire net was covered to prevent the samples from curling (Fig. 1B-B’). The specimens were dehydrated in an ethanol series (50%, 70%, 85%, 95%, 100%, 100%; 20 min per concentration, at 4 °C) and washed twice with propylene oxide (5 min each time, at room temperature). The samples were subsequently treated with mixtures of propylene oxide and epoxy resin (1:1, 1 h; 1:2, 1 h) and then with pure epoxy resin twice for 2 h at room temperature (Fig. 1C).

The procedure for Lowicryl K_4_M resin embedding is as follows. Pre-embedding specimens for immunogold electron microscopy and other samples for TEM were dehydrated in an ethanol series (50% (1 h, − 20 °C), 70% (1 h, − 35 °C), 85% (1 h, − 35 °C), 95% (1 h, − 35 °C), and 100% (1 h, − 35 °C), treated with a mixture of ethanol and Lowicryl K_4_M resin (1:1, 1 h, − 35 °C; 1:2, 1 h, − 35 °C), and then treated with Lowicryl K_4_M resin twice for 24 h at − 35 °C.

### Embedding and polymerizing of samples

The samples for epoxy resin embedding were prepared as follows. Polyvinyl film (approximately 0.2 mm thick) was cut into a large rectangle of approximately 5 × 5 cm and a small, hollow rectangular spacer of approximately 2 × 2 cm and 0.5 mm thick was cut for the bracket. The spacer was then adhered to the large rectangle with A/B glue (Fig. 1D), a drop of epoxy resin was placed inside the spacer, and the sample was placed on the large rectangle with the muscle side up (Fig. 1E). Next, several drops of epoxy resin were placed on the sample, and the spacer and the sample were then covered with polyvinyl film (approximately 3 × 3 cm, 0.2 mm thick) (Fig. 1F). Then the sample was polymerized at 37 °C (24 h), 42 °C (24 h) and 60 °C (24 h). The samples for Lowicryl K_4_M resin were polymerized in UV light with 365 nm wavelength for 3 days at − 35 °C, followed by polymerization for 3 days at 25 °C.

### Trimming and thin sectioning NMJ boutons in the 6th/7th muscles

After removing the large rectangle, the spacer, and the polyvinyl cover, the polymerized sample was a thin slice of approximately 0.5 mm thick, and all the muscles could be observed and located under a light microscope.

Next, an isosceles trapezoid was carved on the sample with a sharp blade (not scissors), retaining the isosceles trapezoid including the A2–A3 segment and 6th/7th muscles, the topline near the 13th muscle, and the baseline away from the sample to epoxy resin. The height between topline and baseline was approximately 5–8 mm (Fig. 1G). The isosceles trapezoid was then removed from the remainder of the sample and placed between a cylinder of cured resin along the baseline, and approximately 1/3 of the trapezoid was adhered with sticky modified acrylate adhesive (A/B glue) (Fig. 1H–I).

**Fig. 2:**
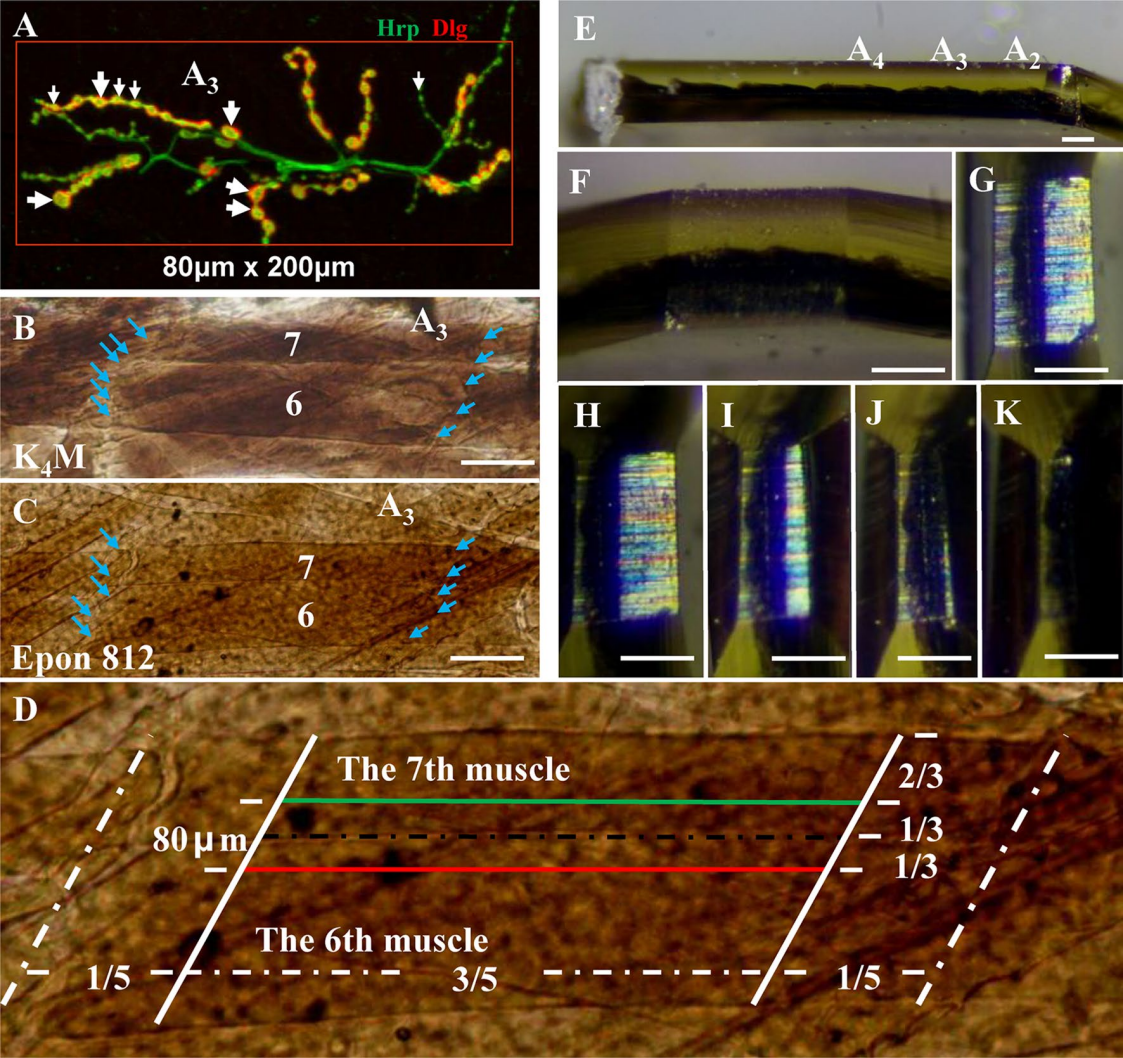
Schematic view of trimming NMJ boutons between the 6th and 7th muscles in *Drosophila* larvae. The type Ib NMJ boutons (big arrow) and type Is NMJ boutons (small arrow) were between the 6th and 7th muscles in *Drosophila* larvae, visualized with immunohistochemistry (**A**), and anti-Hrp antibody labels presynaptic structure of type I NMJ boutons, anti-Dlg antibody labels postsynaptic structure of type I NMJ boutons. The 6th and 7th muscles in *Drosophila* larvae with Lowicryl K_4_M resin (**B**) and Epon 812 resin (**C**). Schematic diagram of continuous ultrathin sections in a parallelogram; the red line indicates the starting position for the ultrathin section, and the green line indicates the end position (**D**). Trim the thin embedding block with a glass knife, and the A_2_, A_3_, and A_4_ body segments are clearly visible (**E**). The A_3_ segment is trimmed and retained (**F**). Excess resin is gradually and carefully removed from the A_3_ segment (**G**–**J**). The cuticle of the body wall is removed (**K**). The blue arrows show the connections between body sections. Scale bar, **B**, **C**: 80 μm; **E**–**K**: 300 μm

The locations of the A_3_ segment (or A_2_ segment) and 6th/7th muscles (Fig. 2A–E) were confirmed, and the tangent plane was maintained parallel to the 6th muscles (Fig. 2E). Then, 1/5 of the sample was cut away along the two sides of the A_3_ segment, most of the 6th muscle was removed, and 1/3 of the width of the 7th muscle was retained (Fig. 2D); thus, the ultrathin slice would start at the 6th muscle.

The excess resin was progressively removed to further reduce the workload (Fig. 2F–J), and the cuticle layer in which there were no NMJ boutons was cut away (Fig. 2K) but the outsides of muscles were not cut (Fig. 2K). Serial sectioning of a total of 80 microns was performed using a diamond knife on a Leica UC7 ultrathin microtome; each slice was 90 nm thick, and 30–40 slices were gathered into a group and attached to a grid.

### Staining and observation

The grids were poststained with 2% saturated uranyl acetate in 50% ethanol and 1% lead citrate (pH 12) and examined under a transmission electron microscope, Hitachi H-7650.

### The procedure for pre-embedding immunogold electron microscopy

Pre-embedding immunogold electron microscopy was performed based on standard procedures (Gan and Zhang 2018). In brief, wandering late 3rd-instar larvae were fixed in a mixed agent (4% formaldehyde, 0.5% glutaraldehyde, and 10% saturated picric acid in 0.1 M sodium cacodylate buffer, pH 7.4) for 4 h, incubated with a mouse anti-Syt (3H2 2D7; 1:5; DSHB) primary antibody (DSHB), and incubated with a 1.4 nm ultrasmall gold-conjugated secondary antibody (goat anti-mouse IgG secondary antibody, Nanoprobes, #2001, 1:50). Then, the silver enhancement (HQS kit; Nanoprobes, #2012) was performed in the dark and the samples were osmicated for 1 h. Subsequent dehydrating and infiltrating, epoxy resin embedding, trimming and thin sectioning were performed as described above for the NMJ boutons at the 6th/7th muscles in the A_3_ or A_2_ segment.

### Immunofluorescence

Immunostaining of the larval samples was performed as described previously (Xing et al. 2014). Briefly, the larval samples were fixed for 40 min in paraformaldehyde, incubated with anti-Hrp (Jackson ImmunoResearch, West Grove, PA) or anti-Dlg (4F3; 1:50; DSHB) at 4 °C for 2 h, and incubated with fluorophore-conjugated secondary antibodies (Invitrogen, 1:500) for 1 h at room temperature. The samples were washed extensively and mounted in VectaShield mounting medium (Vector Laboratories). The images were collected using an Olympus FV3000 confocal microscopy.

## Result

Drosophila larvae were cylindrical, and once the body wall muscle were dissected and fixed with fine needles, they looked thin slice (Fig. 1A). Due to restriction of the metal net, the sample remained flat during the dehydrating process (Fig. 1B, C). The body muscles of the larvae were embedded into a thin sheet with Lowicryl K_4_M resin or epoxy resin (Fig. 1D–G), and it was easy to accurately locate (Fig. 1G) and adhere on the top of capsules that filled with polymerized epoxy resin (Fig. 1H, I).

The NMJ boutons were distributed in a narrow area of approximately 200 × 80 μm between the 6th and 7th muscles (Fig. 2A), so slitting along the muscle could greatly reduce the workload compared with crosscutting (Budnik et al. 1990). The samples became dark brown after fixation with OsO_4_, and the Lowicryl K_4_M resin was white and transparent (Fig. 2B), while the epoxy resin, including Spurr resin (data not shown) and Epon812, was canary yellow (Fig. 2C). The body muscles were sharper and easier to observe with Lowicryl K_4_M resin (Fig. 2B) than with epoxy resin (Fig. 2C), which was more conducive to muscle positioning with Lowicryl K_4_M resin. However, it was easier to perform the procedures with epoxy resin at room temperature, and also permitted precise positioning (Fig. 2C–K).

**Fig. 3:**
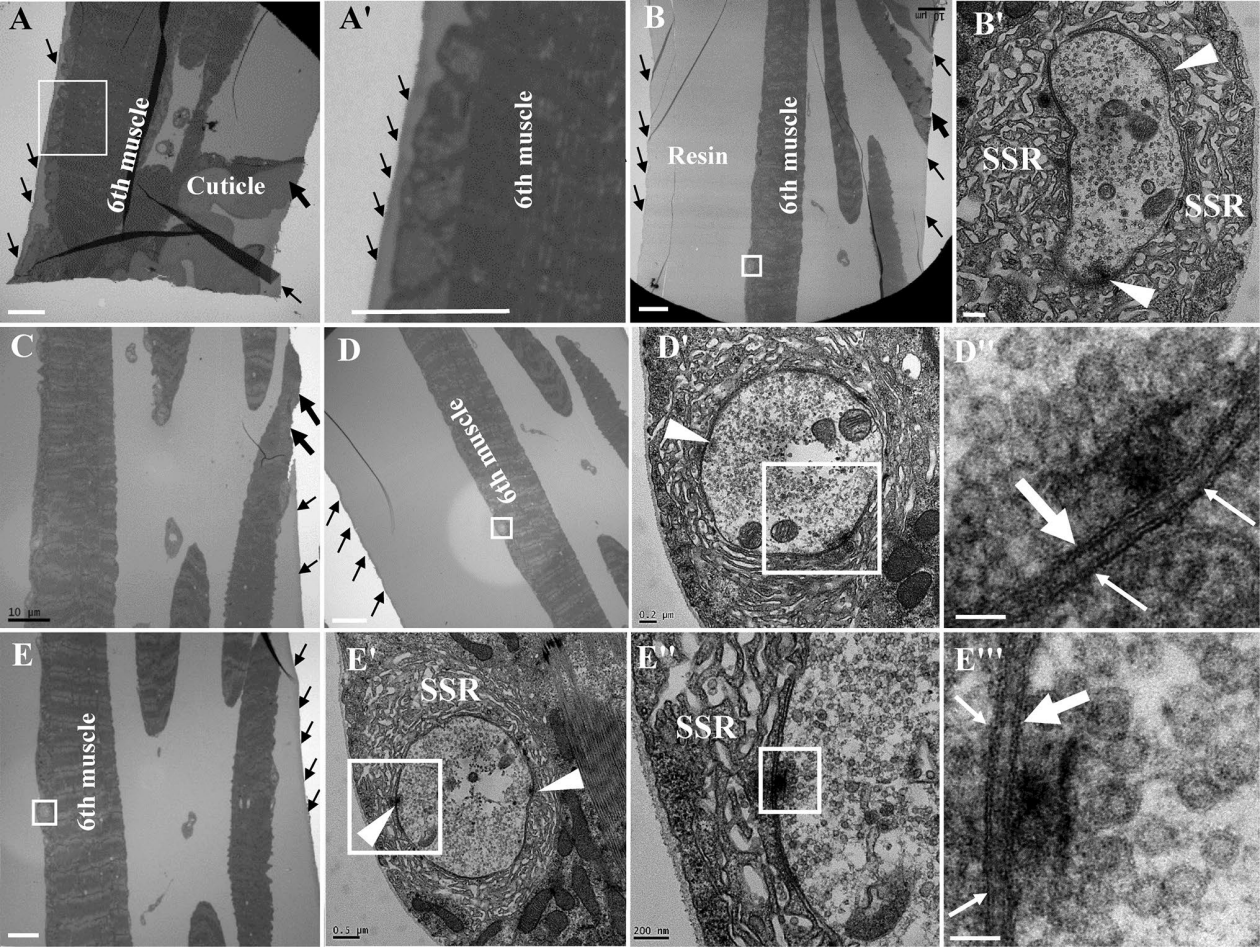
The images and position of NMJ bouton under the electron microscopy. After removing excess resin close to the 6th muscle, and removing the cuticle (**A**, **A’**), thickness of the sample is about 100 microns. As the continuous sectioning progresses, the resin gradually increases (**B**, **D**) and the synapses begin to appear (**B’**). The type Ib boutons were on the surface of the 6th muscle without cuticle on two consecutive slices, and they had dense SSR that can be labeled with anti-Dlg antibody, postsynaptic membrane and presynaptic membrane with T-bar, and clear synaptic vesicles (**D**, **D’’**, **E**, **E’’’**). The thin black arrows show the edges of the trimmed resin, and the thick black arrows show the trimmed muscles and cuticle. The thin white arrows show the postsynaptic membrane, and the thick white arrows show the presynaptic membrane. White wedge show T-bar. **A’**, **B’**, **D’** and **E’** are enlargements of the white box in **A**, **B**, **D** and **E**; **D’’**, **E’’**, and **E’’’** are enlargements of the white box in **D’**, **E’** and **E’’**. Scale bar, **A**, **A’**, **B**, **C**, **D**, **E**: 10 μm; **B’**, **D’**, **E’’**: 200 nm; **E’**: 500 nm; **D’’**, **E’’’**: 50 nm

*Drosophila* larval NMJs were classified as type I, type II, and type III boutons according to their size, subsynaptic reticulum (SSR) characteristics, and synaptic vesicle composition (Atwood et al. 1993; Guangming et al. 2020a; Jia et al. 1993). Removing excess resin near the muscle would effectively reduce the workload (Fig. 2D–K). Did not touch the surface of muscle (Fig. 3A–A’) during trimming, otherwise it might damage NMJ boutons with only about 3–5 microns in diameter. The NMJ boutons were located on the surface of the muscles, but the NMJ boutons might probably not be cut at the beginning of slicing (Fig. 3 A–A’). As the slicing progresses, synapses would appear intermittently on the surface of the muscle (Fig. 3B–B’, D–D’’, E–E’’’) with the increase of resin outside the muscle surface (Fig. 3B, D), and the muscles were gradually covered with resin (Fig. 3B, C, E).

The NMJ boutons could not be observed and positioned under the light microscopy after the preparation of electron microscopy samples, which brought troubles to the precise positioning of the synapse. However, it was easy to search for type Ib boutons with dense SSR and clear synaptic vesicles in the 6th muscle (Fig. 3B–B’, D’, E-E’) or in the 7th muscles with serial sections using the sheet embedding procedure, according to the distribution pattern of NMJ boutons under light microscopy (Fig. 2A) and our recommended ratio of the muscles (Fig. 2D). It did not affect the slicing and observation to remove cuticle (Fig. 3A–A’), on which there is no NMJ bouton, and also effectively reduce the workload.

Due to the irregular distribution of NMJs, NMJs were sometimes not observed (Fig. 3A), sometimes dispersed NMJs could be observed (Fig. 3B, D, E), and sometimes NMJs in the form of beads were observed on a copper net in TEM. Furthermore, in this way, a sufficient amount of typical synaptic structure could be obtained (Fig. 3D-D’, E’-E’’’).

**Fig. 4:**
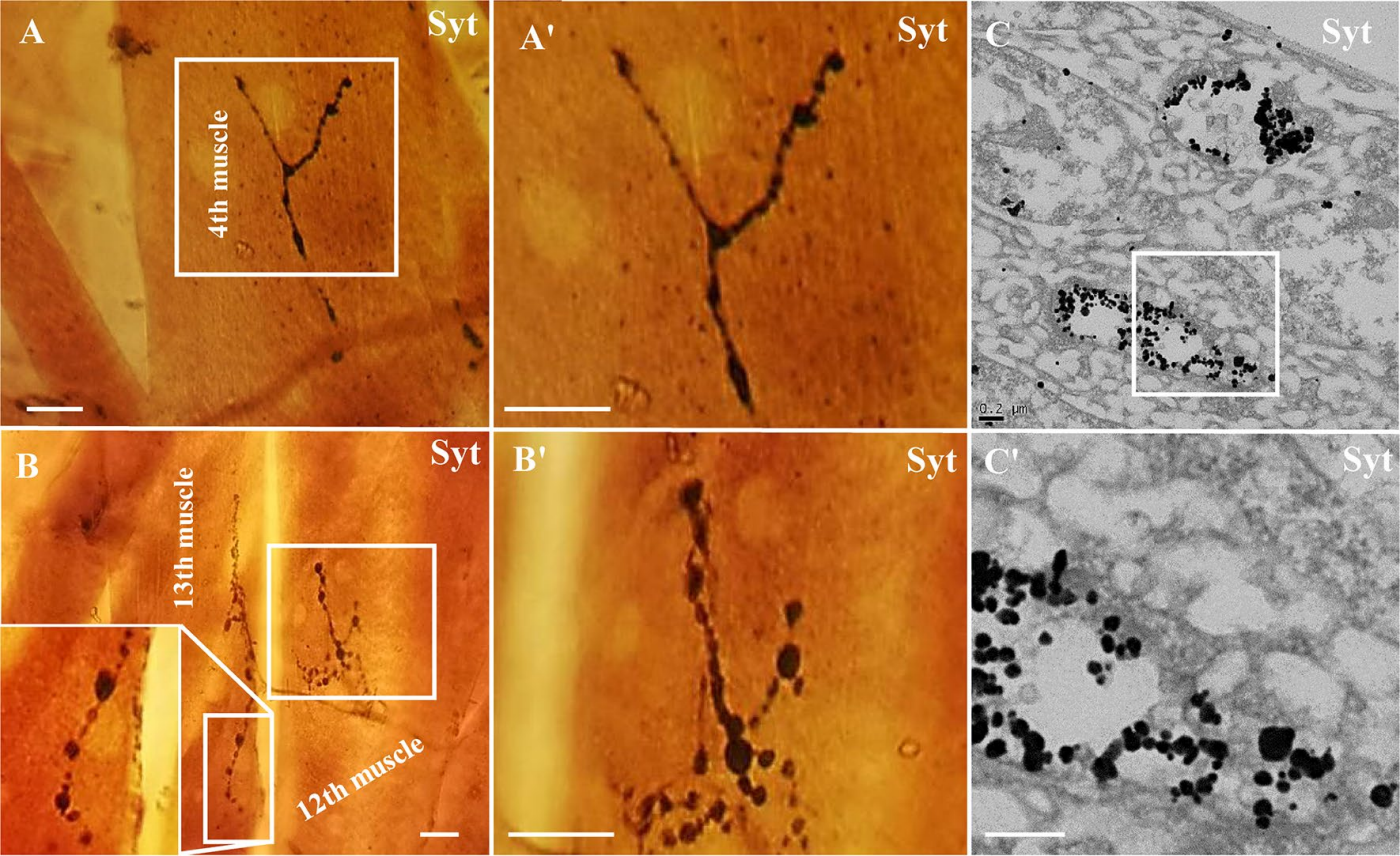
The images and position of immunolabeled NMJ bouton under light microscopy and electron microscopy. The immunolabeled NMJ bouton with Syt (Synaptotagmin) signal in the 4th muscle (**A**, **A’**) and 13th muscle (**B**, **B’**) under light microscopy. The immunolabeled NMJ bouton with Syt signal in 13th muscle (**C**, **C’**) under electron microscopy. **A’**, **B’**, and **C’** are enlargements of the white box in **A**, **B**, and **C**. Scale bar, **A**, **B’**: 10 μm; **C**, **C’**: 200 nm

Immunoelectron microscopy samples of *Drosophila* larval muscle were easier to curl during sample preparation, due to the low concentrations of glutaraldehyde, Tween-20 and saponin which would lightly extract the proteins in the sample. Using the sheet embedding procedure, it was easy to search for localization of immunolabeled NMJ boutons in specific muscles, such as the 4th (Fig. 4A-A’) muscle, the 13th muscle (Fig. 4 B-B’), and observe immune-labeling signal in the NMJ boutons under TEM (Fig. 4C-C’).

## Discussion

### The sheet embedding is helpful for locating muscles and NMJ boutons in Drosophila

The larval specimens were often curl during the dehydration process of preparing sample, which would seriously interfere with the positioning of the NMJ boutons and greatly increase the number of ultrathin sections necessary. If the length of the larva was shortened, the curl of the sample will be weakened (Banerjee et al. 2017), but the bending of the sample is inevitable to different degrees. In our method, the larvae specimens remained flattened between pieces of copper net in a flat-bottomed test tube throughout the dehydration process, whether in ethanol or acetone. With our method, it was easy and clear to position the different muscles and to remove the excess resin and irrelevant muscles as much as we can under light microscopy.

When the muscle sample was polymerizing, it was best to cover it with a smooth covering, otherwise the polymer would form a depression which was not conducive to the positioning of the muscles. Notably, since the cover glass and the slide glass had certain hardness and very flat, the thickness of the cover glass was also conducive to imaging, the most perfect way to observe larval muscles distribution under a light microscope was to place the sheet sample and resin between the glass slide and the cover glass to polymerize. However, this method was not easy to separate the polymerized sample from between the slide and the cover glass because the sample was often tightly attached to the slide glass or the cover glass. In fact, if the sample and the resin were polymerized between plastic products with rough surface, the sample was also not easy to take out due to the close adhesion between the resin and the plastic.

### Proper trimming greatly reduces the workload of section and observation

The type I boutons were distributed over approximately 80 × 200 μm, which was a very large range for ultrathin sections and observation in TEM. Using longitudinal slicing instead of transverse slicing, if each slice was 80 nm, 1000 sections need to be prepared continuously. Therefore, trim the excess resin and muscles without NMJ bouton as much as possible, which could greatly reduce the workload. We recommend mainly the following ways to remove excess resin.

At first, utilize the width of the 7th muscle to trim the 6th muscle. The width of the 7th muscle was less than that of the 6th muscle. According to the method we provided, the thicker 7th muscle could be carefully trimmed gradually, taking the 6th muscle as a reference. When the remaining part of the 6th muscle reached 1/3 of the 7th muscle, the position could be used as the starting point for slicing. When trimming, retaining several body segments at the same time was helped to adjust the parallelism between the tangent plane and the muscle (Fig. 2D), and this process could be done with a sharp glass knife or double-sided blade.

Then remove the two ends of 6/7 muscles. The NMJ boutons were not distributed at both ends of the 6/7 muscles. A recommended empirical value was to remove about 1/5 of the two ends, which can save 40% of the workload. We used to slice the A1–A3 and A2–A3 segments at the same time to get more NMJ boutons under TEM, but due to the sample was too long and narrow, it was not conducive to ultrathin sectioning and collecting the sections on the copper grid. Therefore, it was an efficient strategy to collect enough ultrastructural NMJ boutons between 6 and 7 at single A2 or A3 segment that was commonly used to count the number of NMJ boutons in the conventional literatures under a confocal microscopy.

At last, remove as much resin as possible outside the sample. Generally, to fully polymerize the sample in the resin, a small amount of resin would be left around the sample (Fig. 2E, F). Careful trimming of the excess resin could also reduce the workload exponentially (Fig. 2E–H). The most perfect trimming surface was that the resin was close but did not touch the sample (Fig. 3A-A’), on which surface the NMJ boutons were distributed (Fig. 3B, C). Since the stratum corneum did not have NMJ bouton, it could be trimmed carefully (Fig. 2I–K, Fig. 3A). Finally, the long and narrow section was trimmed, and the thickness of the sample was about 100 microns (Fig. 2K, Fig. 3A).

This method had been applied in *Drosophila* larvae NMJ boutons in our previous research (Sun et al. 2011; Xing et al. 2014; Zhang et al. 2017), and it could significantly reduce the abrasion of diamond knives, the usage of copper grid, and the application of TEM by removing both ends of the 6th/7th muscles, the stratum corneum and its adjacent muscles. Furthermore, this method could also be applied to the small organs such as the *Drosophila* embryo, ventral nerve cord (Gan et al. 2014) and brain (Wu et al. 2017), as well as thin organs such as toads and frog’ flaky lungs (Guangming et al. 2020b) and skins (Guangming et al. 2017) we had done. It should also be applied to the others thin organs such as brain slices in future.
